# Hard-flaccid syndrome: a systematic review of aetiopathophysiology, clinical presentation and management

**DOI:** 10.1038/s41443-025-01118-2

**Published:** 2025-07-07

**Authors:** Karl H. Pang, Jiarong Feng, Yan Zhang

**Affiliations:** 1 Division of Surgery and Interventional Science, University College London, London, UK; 2 Department of Urology, Chelsea and Westminster Hospital NHS Foundation Trust, London, UK; 3 Department of Infertility and Sexual Medicine, The Third Affiliated Hospital of Sun Yat-sen University, Guangzhou, China

**Keywords:** Signs and symptoms, Urogenital diseases, Diagnosis, Pathogenesis, Therapeutics

## Abstract

Hard-flaccid syndrome (HFS) is a rare condition characterised by a semi-rigid penis in the flaccid state, often accompanied by perineal and urinary symptoms. It may also induce psychological distress, which can exacerbate physical symptoms, creating a vicious cycle. There is currently no standardised treatment for HFS, and management typically focuses on addressing both the underlying causes and presenting symptoms. A systematic review of the literature identified 8 eligible studies. Although the exact aetiopathogenesis remains unclear, it is hypothesised that an initial penile trauma may trigger a cascade of neurovascular and inflammatory events. The associated psychological impact may further perpetuate symptoms, reinforcing the cycle of dysfunction. Common symptoms include perineal pain, urinary disturbances, and erectile and ejaculatory dysfunction. Evaluation involves a comprehensive clinical history, relevant blood and radiological investigations to exclude other pathologies, and the use of symptom questionnaires. Reported treatments include phosphodiesterase-5 inhibitors, anxiolytics, low-intensity shockwave therapy, pelvic floor physical therapy, spinal surgery, and biopsychosocial therapy. Management should be individualised, with a focus on relieving symptoms and breaking the self-perpetuating cycle of HFS. Further evidence-based studies are needed to better understand the pathophysiology of HFS, as well as to develop clear diagnostic criteria and management guidelines.

## Introduction

Hard-flaccid syndrome (HFS) is characterised by a semi-rigid penis in the flaccid state which may be associated with other erectile, urinary, ejaculatory, perineal and psychological symptoms [1]. Similar to other male sexual dysfunctions, HFS may cause distress, frustration and interpersonal issues [1]. HFS was first reported and described by Gul et al. following a review of complaints on a hard-flaccid state shared by patients on internet forums and chat groups [2, 3]. The aetiology is not entirely clear but may be related to a trauma-associated event causing injury to the neurovasculature resulting in a complex of erectile, sensory, urinary and musculature symptoms [1]. Goldstein and Komisaruk suggested that HFS associated penile pain is a form of genito-pelvic dysesthesia and may be secondary to pathological activation of a pelvic/pudendal-hypogastric reflex [4, 5].

Due to the rarity of this syndrome and lack of familiarity among clinicians [6], there are no established consensus or guidelines on the diagnostic criteria, workup or management of HFS. Current treatments used include phosphodiesterase-5 inhibitors (PDE5i) with or without low-intensity shockwave therapy (Li-SWT) [2, 7], pelvic floor physical therapy [7–9] and biopsychosocial therapy [8]. Results from a survey demonstrated that the effects of various biopsychosocial interventions are not promising, and patients are not completely satisfied with treatments they received [10]. Current knowledge on HFS arise from case reports, mainly coming from Europe, USA and China. We aimed to perform a systematic review to summarise current perspectives on the aetiopathogenesis, current presentations and how HFS have been managed by other centres who have encountered such cases in order to shed more light on this disorder.

## Methods

The systematic review was registered with PROSPERO (CRD42025634962) and performed with reference to the the Preferred Reporting Items for Systematic Reviews and Meta-Analyses (PRISMA) 2020 guidelines (Supplementary Table 1) [11].

### Study inclusion and exclusion criteria

Our inclusion criteria followed the PICOS framework [12]:

*Population (P)*: men with hard-flaccid syndrome; *Intervention (I)*: any medical or surgical treatment including pharmacotherapy, psychotherapy, physical therapy; *Comparison (C)*: any of the above interventions; *Outcome (O)*: clinical assessment, investigations and treatment outcomes; *Study (S)*: all study and article types including congress abstracts were included. Men without HFS were excluded, as well as articles not in English.

### Search strategy

A search on Medline/PubMed, Embase and Cochrane library using the terms “hard flaccid” OR “hard-flaccid” between 01st January 2018 and 13th April 2025 was performed. We decided to search from 2018 onwards because HFS was initially described at around that time. The titles and abstracts of the articles retrieved from the search were screened for eligibility (KHP and JRF) according to our pre-defined PICOS inclusion criteria. Included abstracts were selected for full-text review. Reference lists of the included full-text articles were also screened for eligible studies.

### Data extraction

Data were collected (KHP and JRF) according to a pro forma which included the author’s name, year of publication, type of study, geographical area, number of patients, clinical signs and symptoms, diagnostic tests performed, interventions and treatment outcomes.

### Data analysis

The risk of bias (RoB) of included studies were assessed (KHP and JRF) using the JBI Critical Appraisal Checklist for case reports, case series and cohort studies [13, 14]. Disagreements between reviewers during study selection, data extraction and RoB assessment were resolved amongst themselves or involving the senior author (YZ). Only a qualitative synthesis of the included studies was possible due to the high heterogeneity of data and the small number of reports on HFS, which hindered any comparative quantitative analysis.

## Results

### Study selection and characteristics

**Fig. 1:**
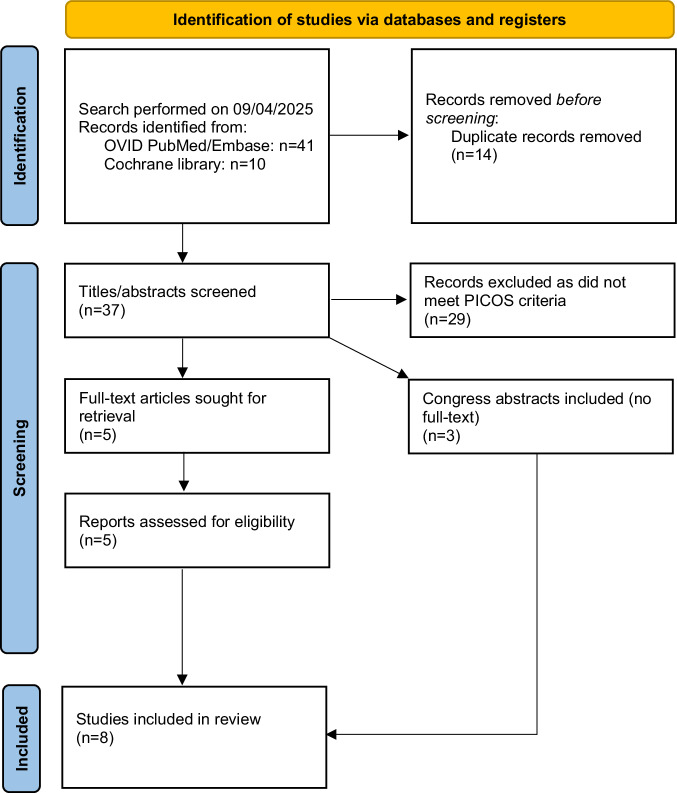
PRISMA 2020 flow chart for the current systematic review.

The study selection is summarised in the PRISMA flow chart (Fig. 1). The initial search identified 51 articles. After removing duplicates, 37 titles/abstracts were screened. Three congress abstracts were included from the first stage of screening and 5 studies were retrieved for full-text screening. Overall, 8 studies were included for data extraction [2, 4, 7–9, 15–17]. There were 2 retrospective studies [16, 17], 1 case series which included 4 patients [2], and 5 case reports [4, 7–9, 15], 1 of which included 2 patients [15].

#### Risk of bias assessment

The results of the RoB assessment of the included studies based on the JBI Critical Appraisal Checklist are summarised in Supplementary Table 2. Most studies were case reports and 3 of the included studies were congress abstracts which resulted in a high risk of bias.

#### Results of individual studies

The study characteristics, and details on patients presenting signs and symptoms, investigations and treatments are summarised in Tables 1–3.

**Table 1:** Baseline characteristics and signs and symptoms.

Author, year	Country	Study type	Number of cases	Age (years)	Duration	Erectile symptoms	Penile symptoms	Ejaculatory symptoms	Perineal/prostate/pelvic symptoms	Urinary symptoms	Psychological symptoms	Other symptoms	Potential cause	Clinical examination
Pang et al. [15]	China	Case report	3	29	4 years (progressed past 4 months)	ED, decreased libido	Penile pain, glans coldness and numbness	No	Perineal pain	Mixed LUTS (frequency, urgency, weak stream, straining)	Anxiety	None	Aggressive masturbation, psychological, RLT	Shrunken penis. Tenderness in penile base. Soft glans
				29	3.5 months	ED, decreased libido	Semi-rigid penis, soft/numb glans	Not ejaculated since onset of symptoms	Penile and perineal “tightness”	None	Anxiety	None	Penile skin abrasion from shaving, psychological, RLT, TEAT	Firm penis, soft glans, dorsal mid shaft scar tissue
Gul et al. [2]	Turkey	Case series	4	34	3 months	ED, decreased libido	Decreased sensitivity and coldness of glans	Painful ejaculation	NR	NR	Anxiety	None	Aggressive masturbation, marijuana	Hardness right base of penis
				26	3 months	ED	Soft and cold glans	NR	NR	NR	Anxiety	None	Trauma during sexual intercourse	Unremarkable
				31	3.5 months	ED	NR	NR	NR	Weak urinary stream	NR	None	Jelqing, aggressive masturbation, history of PD	PD: Tenderness in the proximal penile crura, dorsal penile plaque
				22	2 days	ED, decreased libido	Numb or stinging	NR	NR	NR	NR	None	Jelqing, aggressive masturbation	Unremarkable
Nico et al. [9]	USA	Case report (congress abstract)	1	16	“several months”	“HFS associated symptoms”	Penile and testicular pain and numbness	NR	NR	NR	NR	None	Masturbation	NR
Sullivan et al. [17]	USA	Congress abstract	88	Mean 28 (SD, 12)	Mean 14 (SD, 20)	ED (100%)	NR	NR	NR	NR	66 (75%) depression or anxiety	NR	NR	Hypertonic flaccid penis, normal neurology
Goldstein and Komisaruk, [4]	USA	Case report	1	18	4 months	ED and HFS	Decreased penis and glans sensation. Smaller, firmer, painful flaccid penis	NR	NR	NR	Depression	None	Lumbar disc protrusion with annular tear	Firm flaccid penis, abnormal neurogenital testing
Billis et al. [8]	Greece	Case report	1	30	6 months	ED, decreased libido	Pain in the base of penis, changes in penile shape	None	Perineal pain, coldness and dysethesia, stiffness, coldness and dysethesia in pelvic floor muscles	LUTS: pain during urination, incomplete bladder emptying, increased urinary frequency	Anxious, worried, interrupted sexual and physical activity	None	Intense sexual intercourse	Tightness in abdominal and external perineal muscles on palpation. Tightness and pain in posterior pelvic floor muscles on DRE
Yazar et al. [7]	USA	Case report	1	36	NR	ED, decreased libido	“sensory issues”, penile pain	NR	Perineal and pelvic pain	NR	NR	None	Skin stretching exercises induced pain	Unremarkable
Goldstein et al. [16]	USA	Congress abstract	21	Mean 28 (range 19–42)	NR	NR	NR	NR	NR	NR	NR	38% had low back pain and lower extremity sciatica	Lumbosacral annular tears	NR

*DRE* digital rectal examination; Erectile dysfunction; *HFS* hard-flaccid syndrome, *LUTS* lower urinary tract symptoms, *PD* peyronie’s disease, *NR* not reported, *RLT* red light therapy, *TEAT* thread embedding acupuncture therapy.

**Table 2:** Investigations performed in the individual studies.

Author, year	Blood tests	Penile Doppler USS	MRI	Other tests
Pang et al. [15]	Normal glucose, lipids, FSH, LH, testosterone, prolactin, thyroid function	Normal	Not performed	NPTR: suboptimal erections; urine tests negative; VAS (penile pain): 3–4 at rest and 6–7 during painful attacks; EHS: 2–3/4 (during flaccid); IIEF-5: 11/25 (moderate); IPSS: 21/35 (severe); NIH-CPSI: 34; HADS-A: 10/21 (borderline); HADS-D: 10/21 (borderline)
	Normal glucose, lipids, FSH, LH, testosterone, prolactin, thyroid function	Normal	Not performed	Urine tests negative; VAS (penile pain): 1; EHS: 2–3/4 (during flaccid); IIEF-5: 17/25 (mild); IPSS: 2/35 (mild); NIH-CPSI: 10; HADS-A: 2/21 (normal); HADS-D: 6/21 (normal)
Gul et al. [2]	Normal glucose, lipids, FSH, LH, testosterone	Normal	Normal	NR
	Normal glucose, lipids, FSH, LH, testosterone	Normal	Normal	NR
	Normal testosterone, oestradiol, prolactin	Normal flow, scarring due to PD	NR	NR
	Normal FSH, LH, testosterone, oestradiol, prolactin	Normal	NR	NR
Nico et al. [9]	Normal	Normal	NR	NR
Sullivan et al. [17]	NR	All had normal PSV: Mean 45 (SD,−35) cm/s; 2 (2.3%) had abnormal EDV: Mean 2 (SD, 11) cm/s	NR	NR
Goldstein and Komisaruk, [4]	NR	NR	L5-S1 disc protrusion with annular tear	Neurogenital testing
Billis et al. [8]	Normal	Normal	NR	Transabdominal USS: low pelvic floor muscle mobility; IIEF-5: 18 (mild); urine tests negative; HADS-A: 11 (moderate); HADS-D: 9 (mildly elevated); VAS: 5–6 (deep perineal pain); Pelvic floor muscle strength: 4+ (Oxford scale)
Yazar et al. [7]	Normal testosterone, prolactin, thyroid function	Mild corporal fibrosis	NR	NR
Goldstein, [16]	NR	NR	16/21 (76%) had surgically treatable annular tears, L4-L5 or L5-S1	NR

*EHS* erectile hardness score, *FSH* follicle-stimulating hormone, *HADS* hospital anxiety and depression scale, *IIEF-5* international index for erectile function-5, *IPSS* international prostate symptom score, *LH* luteinizing hormone, *NIH-CPSI* national institutes of health chronic prostatitis symptom index, *NPTR* nocturnal penile tumescence rigidity test, *NR* not reported, *PD* peyronie’s disease, *USS* ultrasound scan, *VAS* visual analogue scale.

**Table 3:** Treatment outcomes and follow-up.

Author, year	Medications	Li-SWT	Physical therapy	Other	Duration (months)	Outcomes
Pang et al. [15]	5 mg Tadalafil, Diclofenac as required, anti-depressants (mirtazapine 15–30 mg once daily, quetiapine 50 mg daily), zopiclone 7.5 mg once daily	Not performed	Not performed	Not performed	1	Improvement in symptoms
	Wuling anxiolytics 330 mg 3× daily	Not performed	Not performed	Not performed	2	Improvement in symptoms. IPSS decreased to 0; NIH-CPSI decreased to 2; VAS, EHS, IIEF-5, HADS did not differ significantly compared to baseline
Gul et al. [2]	5 mg Tadalafil	NR	NR	NR	2	No improvement in symptoms
	5 mg Tadalafil	6 sessions (3600 shocks, 3 Hz, 0.13mJ/mm2)	NR	NR	6	Initial improvement in penile hardness
	5 mg Tadalafil, 50 mg Sildenafil	NR	NR	NR	NR	Some benefit in erections
	50 mg Sildenafil	NR	NR	NR	NR	No improvement in erections
Nico et al. [9]	NR	NR	Pelvic floor PT	NR	NR	Symptom free
Sullivan et al. [17]	91% had/using PDE5i, 45% using anxiolytics or anti-depressants	NR	NR	NR	NR	NR
Goldstein and Komisaruk, [4]	NR	NR	Pelvic floor PT	Sex therapy, left transforaminal epidural spinal injection. Later, left L5-S1 lumbar endoscopic interlaminar discectomy	12	Transient response after epidural injection. Improved erections and penile/glans sensation and reduction in HFS symptoms after discectomy
Billis et al. [8]	5 mg Tadalafil	NR	5 PT within 3-months	Psychological: pain management and coping strategies; Psychosocial: lifestyle and stress-release modifications	3	85% improvement, absent perineal and penile pain/stiffness, IIEF-5: 22 (normal), HADS-A: 7, HADS-D: 6, improved pelvic floor muscle mobility on USS
Yazar et al. [7]	5 mg Tadalafil	6 sessions	PT 1-2× weekly over 10-12w	NR	24	Symptom free
Goldstein, [16]	NR	NR	NR	NR	NR	NR

*EHS* erectile hardness score, *HFS* hard-flaccid syndrome, *HADS* hospital anxiety and depression scale, *IIEF-5* international index for erectile function-5, *IPSS* international prostate symptom score, *LH* luteinizing hormone, *Li-SWT* low-intensity shock wave therapy, *NIH-CPSI* national institutes of health chronic prostatitis symptom index, *NR* not reported, *NSAID* non-steroidal anti-inflammatory drugs, *PT* physical therapy, *USS* ultrasound scan, *VAS* visual analogue scale.

Gul and Serefoglu initially presented 2 cases in a congress abstract [3] and later updated their series to include 2 extra patients resulting in a 4-patient case series [2]. All 4 patients reporting a history of jelqing, aggressive masturbation or trauma during sexual intercourse. Investigations including a penile doppler were normal and all 4 patients were prescribed PDE5i (5 mg tadalafil or 50 mg sildenafil) to manage their symptoms and erectile dysfunction, but this provided temporary or no benefits. One patient received both tadalafil and 6 sessions of Li-SWT with initial relief, however symptoms recurred at 6 months [2].

Nico et al.’s patient was a 16-year-old who develop HFS following masturbation. He was successfully managed with physical therapy. However, the physical therapy protocol and follow-up were not reported [9].

Goldstein and Komisaruk reported a patient who had HFS secondary to a lumbar disc protrusion and was managed definitively with a discectomy. The patient initially presented 4 years prior and conservative treatment including sex therapy and pelvic floor physical therapy were unsuccessful. When the patient revealed a history of back pain and sciatica, a lumber MRI was performed which demonstrated a L5-S1 lumbar disc protrusion with annular tear. Epidural spinal injection provided transient improvement in HFS symptoms, but a L5-S1 lumbar endoscopic interlaminar discectomy provided significant improvement in his HFS symptoms [4]. In a subsequent abstract presentation, Goldstein et al. [16] assessed the prevalence of a lumbosacral annular tear in 21 men with HFS. Overall, 76% had an annular tear, with L5-S1 and L4-L5 being the most common location. In addition, 38% also had concomitant complaints of lower back pain and lower limb sciatica.

Billis et al. reported a more extensive diagnostic workup of a 30-year-old man with HFS secondary to intense sexual intercourse who had 85% symptom improvement at 3 months following biopsychosocial management [8]. The authors evaluated symptoms through validated questionnaires including the International Index for Erectile Function-5 (IIEF-5) and Hospital Anxiety and Depression Scale (HADS) surveys. In addition, abdominal, pelvic floor and perineal muscles were assessed through external palpation and internal digital rectal examination. Pelvic floor muscle mobility was assessed by transabdominal ultrasound scan (USS). Daily 5 mg tadalafil was initiated together with 5 physical therapies, pain management and coping strategies, lifestyle and stress-release modifications. At 3 months, there were improvements in IIEF-5 and HADS scores and pelvic floor muscle mobility on USS. In addition, perineal and penile pain/stiffness were no longer present, however, the patient still had stress-related response issues and sexual performance anxiety [8].

The case with the longest follow-up was reported by Yazar et al. The 36-year-old patient was treated with trimodality therapy incorporating daily 5 mg tadalafil, 6 sessions of Li-SWT and 1–2 weekly physical therapy over 10–12 weeks. At 24 months, the patient remained symptoms free [7].

Pang et al. [15] presented 2 cases, both of which experienced some form of penile trauma either by aggressive masturbation, thread embedding acupuncture therapy (TEAT) or red light therapy (RLT). Similar to Billis et al., they used IIEF-5, HADS as well as erectile hardness score (EHS), International Prostate Symptom Score (IPSS) and the National Institutes of Health Chronic Prostatitis Symptom (NIH-CPSI) survey to evaluate the patients’ symptoms. Blood tests and colour doppler ultrasound (CDUS) were normal. One patient had a nocturnal penile tumescence rigidity (NPTR) scan, which showed suboptimal erections. Both patients were offered anxiolytics, analgesia and tadalafil as required and patient-1 was on anti-depressants prescribed by his psychiatrist. At a follow-up of 1–2 months, both patients claimed that their symptoms improved. Moreover, patient-2 had vast improvements in his IPSS and NIH-CPSI scores (Table 3).

The erectile haemodynamics were evaluated in 88 patients with HFS by Sullivan et al. [17]. Seventy-five percentage (*n* = 66) of these patients also had a history of depression or anxiety and 45.5% (*n* = 40) were actively using anxiolytics or anti-depressants. On penile CDUS, all patients had normal peak systolic velocity (PSV), 2 (2.3%) patient had an abnormal end diastolic velocity (EDV) and no patient had elevated echogenicity on B-mode scanning.

## Discussion

In this systematic review, we provided an update on the current clinical presentation and management of patients with HFS.

### Clinical presentation

From the current systematic review, men diagnosed with HFS are aged between 16–42 years old. In the studies that reported potential aetiologies, all patients had a history of some form of trauma including aggressive masturbation, intense sexual intercourse, lumbar disc prolapse, annular tears, penile skin stretching/injury and possibly TEAT and RLT. It is evident that HFS associated symptoms varied across all patients and there was no standardised reporting of symptoms as demonstrated in Table 4. It is important to standardise reporting of symptoms in order for data to be compared in future studies to avoid heterogenicity. Since there are no agreed diagnostic criteria, it is appropriate to use the clinical features “list” described by Gul et al. as it represents the commonest symptoms identified from patient forums [1, 2, 18].

**Table 4:** Comparison of symptoms and assessment amongst included studies according to Gul et al.’s [2] suggested clinical features list.

		Pang et al. [15]		Gul et al. [2]				Nico et al. [9]	Sullivan et al. [17]	Goldstein and Komisaruk, [4]	Billis et al. [8]	Yazar et al. [7]	Goldstein, [16]
Symptoms of HFS		*n* = 2		*n* = 4				*n* = 1	*n* = 88	*n* = 1	*n* = 1	*n* = 1	*n* = 21
Penis	Feels constantly hard but in flaccid state	Y	Y	Y	Y	Y	X	Y	Y	Y	Y	Y	Y
	During masturbation, slight ache in the base of the penis	Y	NA (not ejaculated)	Y	Y	Y	Y	NR	NR	NR	NR	NR	NR
	Noticeable superficial veins	N	Y	Y	Y	N	N	NR	NR	NR	NR	NR	NR
	Bubble around the glans (very rare)	N	N	N	Y	N	N	NR	NR	NR	NR	NR	NR
	Scar tissue (very rare)	Y	N	Y	N	N	N	NR	NR	NR	NR	NR	NR
Erections	No morning erections	N (reduced morning erections)	N (reduced morning erections)	Y	Y	Y	Y	NR	NR	NR	NR	NR	NR
	Often feel hollow or empty but also rigid than usual	Y	Y	Y	Y	Y	Y	NR	NR	NR	NR	NR	NR
	Glans is often soft, sometimes cold or numb	Y	Y	Y	Y	N	Y	NR	NR	Y	NR	NR	NR
	Difficult to maintain erections	Y	Y	Y	Y	N	Y	NR	Y	Y	NR	Y	NR
	Best in lying on back position, worst when stood upright	N (worse lying)	Y (worse stood up)	Y	N (opposite)	Y	N	NR	NR	NR	NR	NR	NR
Libido	Generally low	Y	Y	Y	Y	N	Y	NR	NR	NR	Y	Y	NR
Urination	Painful urination	Y	N	N	N	N	N	NR	NR	NR	Y	NR	NR
	Weak stream (rare)	Y	N	N	N	Y	N	NR	NR	NR	N	NR	NR
Ejaculation	Painful ejaculation (or slightly painful)	N	NA (not ejaculated)	Y	Y	Y	Y	NR	NR	NR	N	NR	NR
Pain	Penile and/or perineal (occasionally)	Y	Y (tightness)	N	N	N	N	Y	NR	NR	Y	Y	NR
Examination and tests	Normal physical examinations, sometimes mild curvatures	N (tender penis)	N (soft glans)	Y	Y	Y	Y	Y	Y	N (smaller, firmer, painful)	Y	Y	NR
	Generally normal hormone levels and other blood tests	Y	Y	Y	Y	Y	Y	Y	NR	NR	Y	Y	NR
	Normal penile doppler ultrasonography (no Peyronie’s, no fibrosis)	Y	Y	Y	Y	N (stable PD)	Y	Y	NR	NR	Y	Y	NR
	Normal MRI and other imaging modalities	N (abnormal RigiScan)	NA (not performed)	Y	Y	Y	Y	NR	NR	N (lumbar disc protrusion)	N (pelvic floor muscle stiffness on USS)	NR	N (76% annular tears)

*Y* yes, *N* no, *NA* not applicable, *NR* not reported.

A survey distributed at the 2023 American Urological Association (AUA) meeting received 36 responses and nearly a third of participants had never seen HFS in their practice and about half of the respondents who had encountered HFS were confident in its legitimacy as a real medical syndrome. This survey highlighted the ongoing lack of familiarity [6].

HFS includes a cluster of symptoms reported by patients on the internet. The suggested diagnostic features were developed through the qualitative analysis of internet forum discussions on HFS [2, 18]. There are currently no objective tests to help diagnose HFS as the aetiology and pathophysiology is not entirely clear, and the diagnosis is mainly based on subjective symptoms review and exclusion of other pathologies through blood and radiological tests. A recent survey conducted by Niedenfuehr and Stevens on HFS distributed on social media platforms received 143 responses [10]. The mean age of the participants in the survey was 27.4 years confirming that HFS predominantly affects young men. The authors presented a more extensive list of symptoms compared to Gul et al.’s [2], and the most common symptoms (experienced by >65% of patients) were: changes in penis shape/size (92.3%), rigid penis when not erect (90.9%), psychological distress, anxiety and/or depression (89.5%), weak, tight and/or overactive pelvic muscles (85.3%), numbness/loss of sensation anywhere on the penis (74.8%), difficulty or inability to have an erection (74.1%), decreased force of urinary stream (72.7%), changes in dorsal vein size (71.8%), cold glans (66.7%) and loss of morning erections (66.2%) [10]. Since the most common symptoms apart from having a rigid penis when not erect were changes in penile shape/size and psychological symptoms, it is appropriate to include these 2 symptoms to Gul et al.’s [2] “list” as both of our patients had psychological distress, 1 of which also reported a change in penile size. In addition, in Sullivan et al.’s study 75% of patients had a history of depression or anxiety [17].

### Aetiology and pathophysiology

The onset of symptoms was hypothesised to arise from some form of minor trauma [1, 2, 19]. However, in Niedenfuehr and Stevens HFS survey, 58% claimed that their HFS symptoms appeared following an incident or injury [10]. Therefore, HFS could possibly be idiopathic in origin. In Pang et al.’s [15] report, a patient received penile TEAT and both patients underwent RLT in the private sector for which the indication and therapeutic benefits were not entirely clear. TEAT involves inserting biodegradable sutures into acupoints, usually subcutaneously. It is practiced in Korean and Traditional Chinese Medicine with the aim to provide continuous stimulation of the acupoint avoiding regular acupuncture visits [20, 21]. Randomised-controlled trials have demonstrated that TEAT is effective in managing musculoskeletal pain such as osteoarthritic pain [22], or low back pain [23, 24] and abdominal obesity [25]. In addition, penile TEAT has been demonstrated to be effective in managing premature ejaculation [26]. However, there are currently no published clinical data on the role of TEAT in managing erectile dysfunction. In rat models with cavernous nerve injury, it has been shown that red-light controllable nitric oxide releaser, NORD-1 with red-light irradiation improved erectile function [27]. However, its role in human erectile dysfunction is unknown as there have been no clinical studies on human evaluating this.

The hypothesis on the aetiology and pathophysiology of HFS includes the initiation of inflammation following a trauma-like event involving the pudendal nerve and/or vasculature inducing neuropathy, penile hypoxia and muscle spasms. These muscle spasms may increase the intracavernosal pressuring during the flaccid phase of erection and inhibit optimal erection during the rigid phase, causing a hard-flaccid penis. In addition, the muscle spasms may also be associated with symptoms seen in chronic pelvic pain and primary prostatic pain syndromes. The neuropathy and penile hypoxia may cause the coldness and numbness in the glans and penile shaft reported by patients. The symptom complex may induce anxiety and distress and in turn worsen muscle spasms and symptoms resulting in a vicious circle between psychological ad HFS symptoms [1, 2, 18].

Interestingly, Goldstein and Komisaruk hypothesised that HFS is a result of pathological activation of a somato-visceral and/or a viscero-visceral reflex that they termed a “pelvic/pudendal-hypogastric” reflex [4]. This reflex may be pathologically activated via triggers located in 5 regions: (1) end organ (penis); (2) pelvis/perineum; (3) cauda equina; (4) spinal cord; (5) brain. Any insult at these levels, for example penile injury (e.g. aggressive masturbation) or pelvic/perineum injury would result in excess sympathetic activity and penile and pelvic/perineal symptoms respectively. Symptoms relief may be obtained by down-regulating the sympathetic drive, for example anti-inflammatory medications or Li-SWT for penile symptoms, and muscle-relaxants or pelvic floor physical therapy for pelvic/perineal symptoms [4]. In Goldstein and Komisaruk’s case, the patient had a disc prolapse resulting in injury in “region 3” and subsequent lumber discectomy resulted in significant relief of symptoms. Whilst this hypothesis is intriguing and logical, more research and patient cases are required to test this. In addition, Goldstein et al. identified that in 21 men with HFS and sacral radiculopathy, 16 (76%) had a surgically treatable annular tear [16]. Further follow-up regarding whether surgery was performed and whether symptoms resolved were unknown.

In Pang et al.’s [15] cases, psychotropic medications were given to both patients, and symptoms appeared to have improved, further suggesting a psychological component to HFS. In irritable bowel syndrome the gut-brain-axis represents a complex communication network between the gastrointestinal tract and the central nervous system. In individuals with irritable bowel syndrome, this system is believed to be dysregulated, resulting in atypical responses to stress, emotions, and gastrointestinal function [28]. Similarly, in the case of HFS, there may be a comparable “penis-brain-axis” involved in the manifestation of symptoms. However, this remains a hypothesis that requires further scientific investigation. Given the relatively mild degree of injury in these two patients, it cannot be ruled out that the trauma may have triggered the onset of HFS by inducing psychological abnormalities in individuals with an underlying psychological vulnerability.

### Clinical assessment and investigations

Most studies in this review performed baseline bloods including hormonal profile to rule out organic cause of the patients’ erectile symptoms. In addition, most studies included at least an USS, not to diagnose HFS as such, but to rule out any abnormal blood flow or penile masses that may explain the patients’ penile symptoms. Therefore, in patients presenting with HFS, initial baseline blood tests and USS are suggested to exclude any differential diagnoses. Sullivan et al. reported that all patients in their study who had a penile CDUS, all had normal PSV and only 2 (2.4%) patient had an abnormal EDV [17].

Apart from identifying a list of signs and symptoms from patients’ history, evaluation of the degree and impact of symptoms through relevant questionnaires or scoring aids may be useful. Billis et al. used VAS, IIEF-5 and HAD questionnaires [8], and Pang et al. [15] used IIEF-5, EHS, VAS for pain, IPSS, NIH-CPSI and HADS. Using these questionnaires, it was evident that, patient 1 had more symptoms of higher severity compared with patient 2 in their report [15]. In addition, both patients claimed that their symptoms had significant impact on the quality of life (QoL) this suggested that the degree of impact on QoL may not necessarily correlate with the severity of symptoms [15].

Pang et al. [15] also utilised NPTR to objectively assess penile tumescence and rigidity. Although this test may not be readily available outside specialised centres and may offer limited diagnostic value, it can be useful for evaluating underlying psychogenic erectile dysfunction when no clear cause is identified through biochemical or radiological investigations. However, its accuracy can be influenced by the patient’s sleep state.

If patients present with HFS associated with back pain, sciatica or signs of radiculopathy, a lumber spine MRI may be required to rule out any spinal pathology as demonstrated in Goldstein at al’s reports [4, 16].

### Management and outcomes

Various treatment and outcomes were identified in this review which included PDE5i, Li-SWT, physical therapy and lumber spine surgery. In the reported cases, only 2 (20%) patients were symptom free, 1 patient following physical therapy [9], and the other patient following trimodal therapy with PDE5i, Li-SWT and physical therapy [7]. It is likely that patients require multimodal therapy to target different physical symptoms accordingly as suggested by Goldstein and Komisaruk [4]. In addition psychotherapy or anxiolytics/anti-depressants should be consider appropriately to break the vicious cycle of HFS. Billis et al. used a combination of biopsychosocial therapy and their patient reported 85% improvement in symptoms [8]. In addition Pang et al. [15] also reported improvement in symptoms with both patients following a course of anti-depressants or anxiolytics.

In Niedenfuehr and Stevens’s survey, treatments patients received included PDE5i, pelvic floor physical therapy, SWT, diet/nutrition changes, nerve blocks, muscle relaxants, anti-inflammatory medications, cognitive therapy and nerve pain medications. No treatments provided significant improvements or complete cure. PDE5i was perceived the most efficacious, with patients reporting between “little” to “moderate” improvement. The other treatments provided “no” to “little” improvement. In addition, no patients were completely satisfied with any of the treatments and PDE5i received the highest satisfaction score (mean 4.8 on an 11-point slider scale) [10].

It appears that, most treatments do not provide complete cure, and patients are commonly not satisfied with the treatments they received. HFS is a complex disease to manage, and treatment would require multimodal therapy via a multidisciplinary approach and should be personalised according to the patient’s presenting symptoms. Treatments may not result in a cure unless the aetiological factor is eliminated, but more to relief symptoms and break the vicious cycle of HFS and may require coping mechanism to focus on factors that relieve symptoms and to avoid factors that exacerbate symptoms.

### Limitations

The limitation of this systematic review is the small number of patients and heterogenicity in data reporting which restricted any quantitative analysis. However, HFS is extremely rare and may be under-diagnosed or reported by clinicians due to the unfamiliarity. Larger case series are required in the medical literature to allow continued education to improve familiarity of HFS, and in order to increase the case load enabling more meaningful analysis in the future.

### Implications of results

This systematic review has highlighted the range of symptoms associated with HFS, the differences in clinical assessment tools used and the different treatment approaches. In addition, this review summarised the current hypothesis suggested by experts in the field. Due to the heterogenicity in data reporting, lack of clinical guidelines on diagnostic workup and management, and lack of familiarity in general amongst clinicians, it is imperative that an expert consensus recommendation on HFS is developed. In addition, apart from the clinical aspects, future research in the basic science of HFS may unravel molecular mechanisms associated with the pathophysiology of this syndrome, allowing the investigation into therapeutic agents.

## Conclusion

The aetiopathophysiology of HFS is not entirely clear but involves complex neurovascular pathways. Investigations should aim at ruling out any sinister pathologies which may explain the patient’s symptoms. Treatment usually requires multimodal therapy targeting physical and psychological symptoms.

## Supplementary information

Supplemental Material 1-2

## Data Availability

All data generated or analysed during this study are included in this published article.
